# First biological measurements of deep-sea corals from the Red Sea

**DOI:** 10.1038/srep02802

**Published:** 2013-10-03

**Authors:** C. Roder, M. L. Berumen, J. Bouwmeester, E. Papathanassiou, A. Al-Suwailem, C. R. Voolstra

**Affiliations:** 1 Red Sea Research Center, King Abdullah University of Science and Technology, Thuwal, Saudi Arabia; 2 Biology Department, Woods Hole Oceanographic Institution, Woods Hole, USA; 3 Hellenic Centre for Marine Research, Anavissos, Greece; 4 Coastal and Marine Resources Core Lab, King Abdullah University of Science and Technology, Thuwal, Saudi Arabia

## Abstract

It is usually assumed that metabolic constraints restrict deep-sea corals to cold-water habitats, with ‘deep-sea’ and ‘cold-water’ corals often used as synonymous. Here we report on the first measurements of biological characters of deep-sea corals from the central Red Sea, where they occur at temperatures exceeding 20°C in highly oligotrophic and oxygen-limited waters. Low respiration rates, low calcification rates, and minimized tissue cover indicate that a reduced metabolism is one of the key adaptations to prevailing environmental conditions. We investigated four sites and encountered six species of which at least two appear to be undescribed. One species is previously reported from the Red Sea but occurs in deep cold waters outside the Red Sea raising interesting questions about presumed environmental constraints for other deep-sea corals. Our findings suggest that the present understanding of deep-sea coral persistence and resilience needs to be revisited.

Although first observed more than a century ago^1^, coral ecosystems of the deeper aphotic zones of the oceans have only recently attracted broad interest^2^. Deep-sea corals primarily inhabit the dysphotic and aphotic zones of continental shelves, mainly in areas of distinct topography such as slopes, canyons, and seamounts^3^. Relatively strong currents in such areas maximize the food supply^4^ and also facilitate sediment removal from either sessile organisms or the seafloor to provide suitable settlement substrata for coral larvae^5^. In addition to nutrient availability, the main factors determining coral settlement include temperatures not exceeding 12°C^2^,^3^,^6^, aragonite saturation^7^, and sufficient oxygen levels^8^. While the latter two are vital to maintaining calcification and aerobic metabolism in corals, the low temperature regimes decelerate food decay and reduce metabolic demands. Aphotic deep-sea corals are thus synonymously referred to as ‘cold-water corals’^e.g.^ 2,3,4,6,7.

Deep-sea coral reefs are largely valued for their importance as biodiversity hotspots^2^, climate archives^9^, habitats for commercial fish species^10^, and promising sources of bioactive compounds^11^. To date, most research in the area has focused on describing the diversity and distribution of deep-sea coral reefs. While responses to local disturbances such as deep-sea trawling, seafloor drilling, mining, or oil spills have been investigated^12^, the impacts of global climate change, such as ocean acidification and warming, remain less well understood, especially because they are difficult to measure in deep-water habitats. An excess of dissolved carbon dioxide in the oceans has been predicted to shoal the aragonite saturation horizon (ASH), limiting the availability of suitable habitat for deep-sea corals^13^. By 2099 more than two thirds of the known deep-water coral habitats are predicted to be located in waters below the ASH potentially altering calcification abilities of corals, and possibly impeding planktonic growth, and hence affecting food availability for the deep-sea benthos^14^. Rising sea surface temperatures, a well-described threat to shallow-water coral reefs resulting in bleaching and mass mortalities^15^, have been documented at great depths^16^. Although deep-sea corals can tolerate substantial fluctuations in temperature^17^, a rise of 2°C for only a few hours can cause significant increases in energy demands^18^ with potentially serious consequences for organisms thriving in nutrient-deprived environments. A detailed understanding of the impacts of global climate change on the biology of slow-growing, long-living deep-sea corals is therefore critical.

The Red Sea is one of the warmest, most saline, and most oligotrophic marine ecosystems on Earth. It is unique in sustaining year-round temperatures exceeding 20°C throughout the water column (>2000 m)^19^. Unfortunately, the ecology of the Red Sea is under-studied, particularly outside of the Gulf of Aqaba^20^. Nonetheless, it is known for its extensive shallow water coral reefs of which some species are known to occur in depths outside the photic zone, in areas where available light is below 1% of that from the surface^21^. The presence of azooxanthellate corals in deep waters (between 400 – 1000 m) of the Red Sea was first documented in the late nineteenth century^22^, but it was not possible to collect biological measurements at that time. Later work utilized extensive submersible deployments in the Gulf of Aqaba^21^,^23^,^24^ to investigate extreme boundaries of the mesophotic reef system (to approximately 200 m depth). To date, however, no mechanism has been provided to explain the persistence of azooxanthellate corals at depths between 200 – 1000 m in the Red Sea under relatively warm water temperatures, nor has their physiology and metabolism been studied. Because the existing body of knowledge of deep-sea coral is based on studies of cold-water corals, the identification and study of deep-sea corals and their underlying biology in the Red Sea could open the door to new studies of the mechanisms used to adapt to these unique conditions.

The overall aim of this study is to present a report of opportunistic sampling of deep-sea corals in the Red Sea. We give some description of the distribution and habitat of various species of the observed deep-sea corals. Measurement of some basic biological characters provides some insight to how these corals function and survive in such a unique environment. These results are particularly interesting in light of current theories of deep-sea coral biology, which assume much cooler water conditions.

## Results

### Study sites

All four sites investigated by ROV or submersible were found to have corals present (Table 1). Two sites were found to have multiple species of corals, while two sites had only single species present (although sometimes in large numbers). All four sites showed high topographic relief (seamount-like or steep-walled habitats) (Supplement Fig. 1).

### Environment of the deep Red Sea

**Figure 1:**
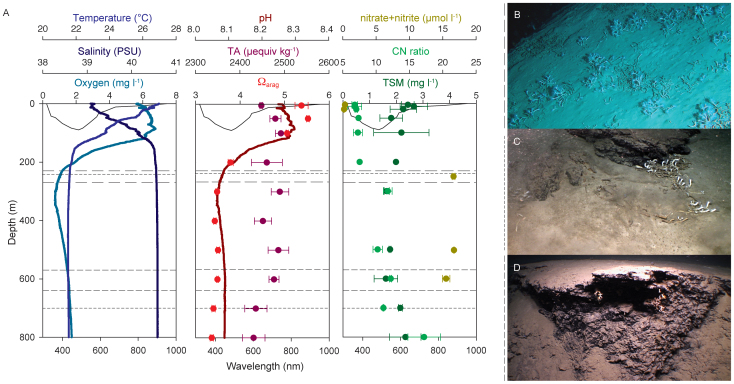
(A) Depth profiles (y-axes) of physical and biogeochemical water properties of the Red Sea. Left graph: temperature, salinity, oxygen concentration. Middle graph: pH, total alkalinity (TA), aragonite saturation (Ω_arag_). Right graph: nitrate plus nitrite concentrations, carbon-to-nitrogen ratios, total suspended matter (TSM) contents. Solid black lines in each graph indicate the margin of the photic zone (1% surface radiation) for all wavelengths within the spectral photosynthetically active radiation (PAR) range. Differently dashed lines represent distribution depths of the three sampled coral species: the style of the dashed line indicates the respective coral species in its preferred habitat ((B) *Eguchipsammia fistula*; (C) Species A; (D) *Dendrophyllia* sp.). Images obtained with ROV camera.

While temperature in the aphotic zone of the Red Sea barely varies from 21.5°C and salinity below the thermocline increases to more than 40 PSU, oxygen concentrations were found to be lower than 2 mg l^−1^ in subthermocline water (> 200 m depth), and at depths around 300 m declined even below 1 mg l^−1^ (Fig. 1). With a pH between 8–8.1 below the thermocline and a total alkalinity (TA) between 2400–2500 μequiv kg^−1^, aragonite saturation (Ω_arag_) in the sampling sites was on average 3.5 (Fig. 1). Nitrate plus nitrite concentrations rose from almost 0 in surface to more than 15 μM in deep water. While amounts of total suspended matter (TSM) were relatively consistent over the water column, C:N ratios increased with depth (Fig. 1). Specific measurements of environmental parameters were taken at two sites where coral samples were collected (Table 1); these measurements were consistent with the results from our profiling of the water column (Fig. 1).

### Taxonomy

At least six deep-sea coral species were observed in the Red Sea of which the following three were successfully collected and morphologically examined.

**Figure 2:**
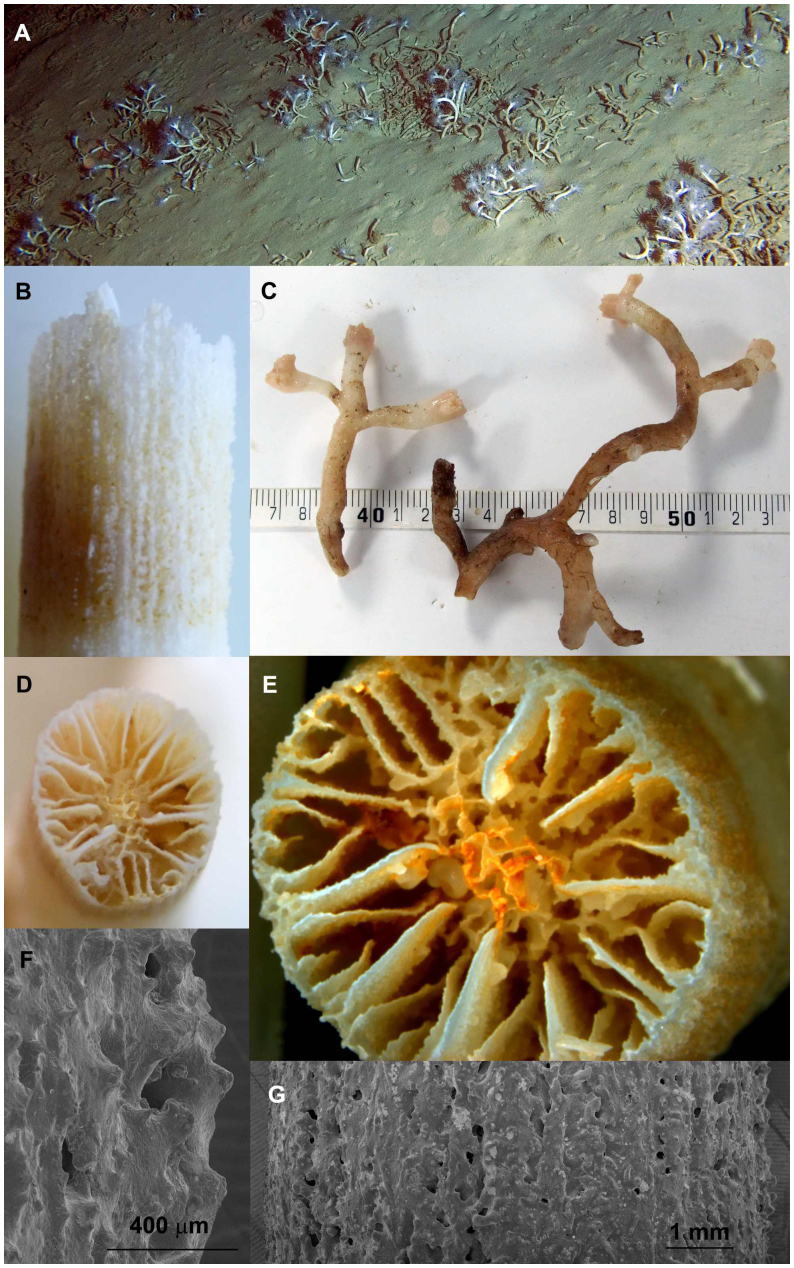
*Eguchipsammia fistula*, Dendrophylliidae. (a) Habitat view. (b) Lateral view of corallum. (c) Colony view (scalebar in cm). (d) and (e) Calicular view of corallum. (f) and (g) Close-up (SEM) of the theca half way up the corallum. Image a obtained with ROV camera. Image c by Roder/Voolstra. Images b, d, e, f, g by Bouwmeester.

*Eguchipsammia fistula* (Dendrophyllidae) (Fig. 2): Corallum colonial, corallum and septa porous, branching extra-tentacular, septa arranged in Pourtalès plan, columella spongy, theca porous, no pali. Colonies formed monospecific coral gardens on seamount tops. In some cases, gardens of dead skeleton were also found on the seamounts, usually a few meters below the top.

**Figure 3:**
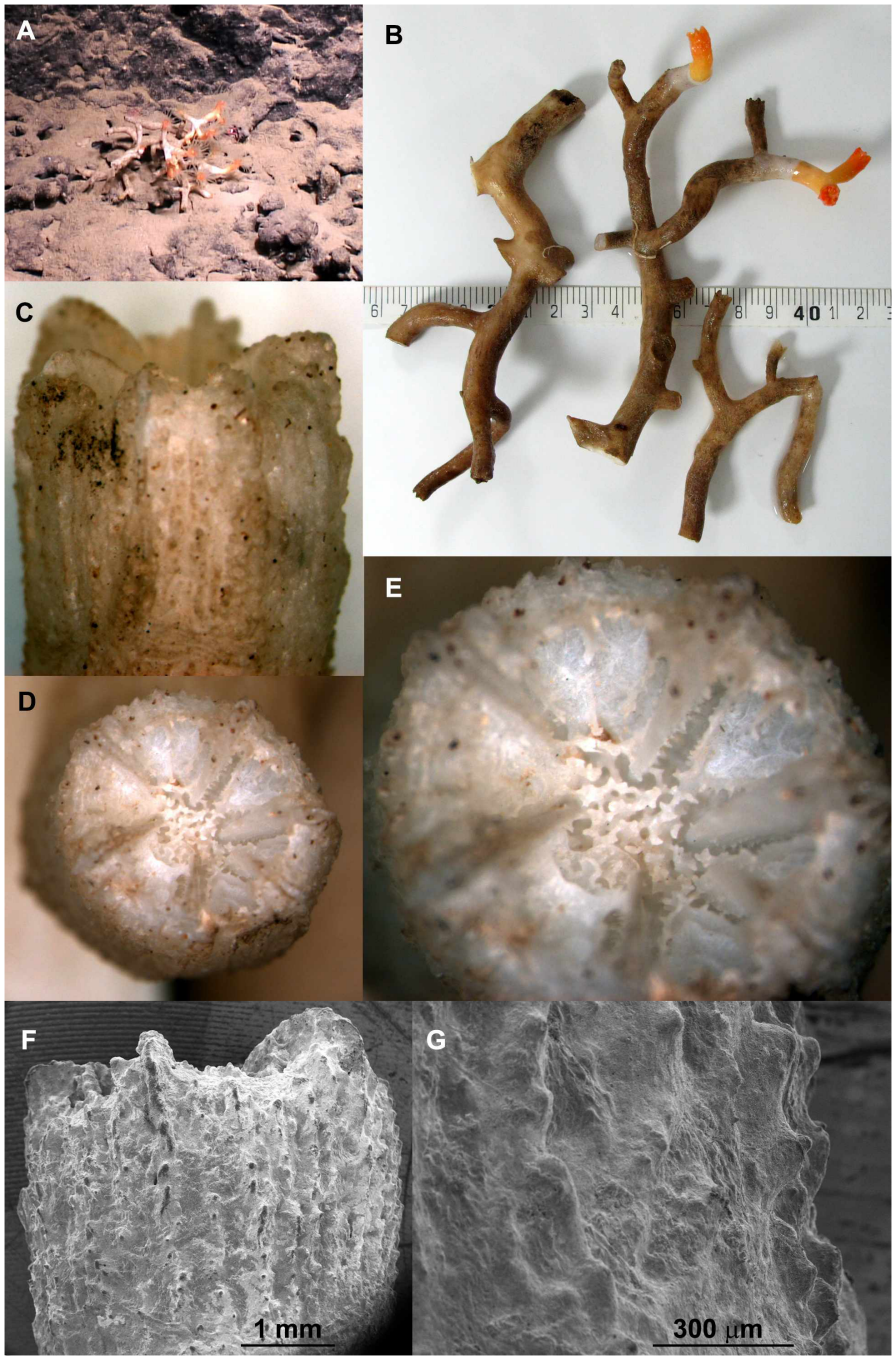
*Dendrophyllia* sp, Dendrophylliidae. (a) Habitat view. (b) Colony view (scalebar in cm). (c) Lateral view of corallum. (d) and (e) Calicular view of corallum. (f) Close-up (SEM) of the theca at the top of the corallum. (g) Close-up (SEM) of the microstructure of the theca. Image a obtained with ROV camera. Image b by Roder/Voolstra. Images c, d, e, f, g by Bouwmeester.

*Dendrophyllia* sp. (Dendrophyllidae) (Fig. 3): Corallum colonial, branching extra-tentacular, septal symmetry hexameral, columella spongy, theca rough, single cycle of septa observed on sample, no pali. Colonies were found around ridges, mainly hanging under a ridge but also in compacted sand just below or above a ridge.

**Figure 4:**
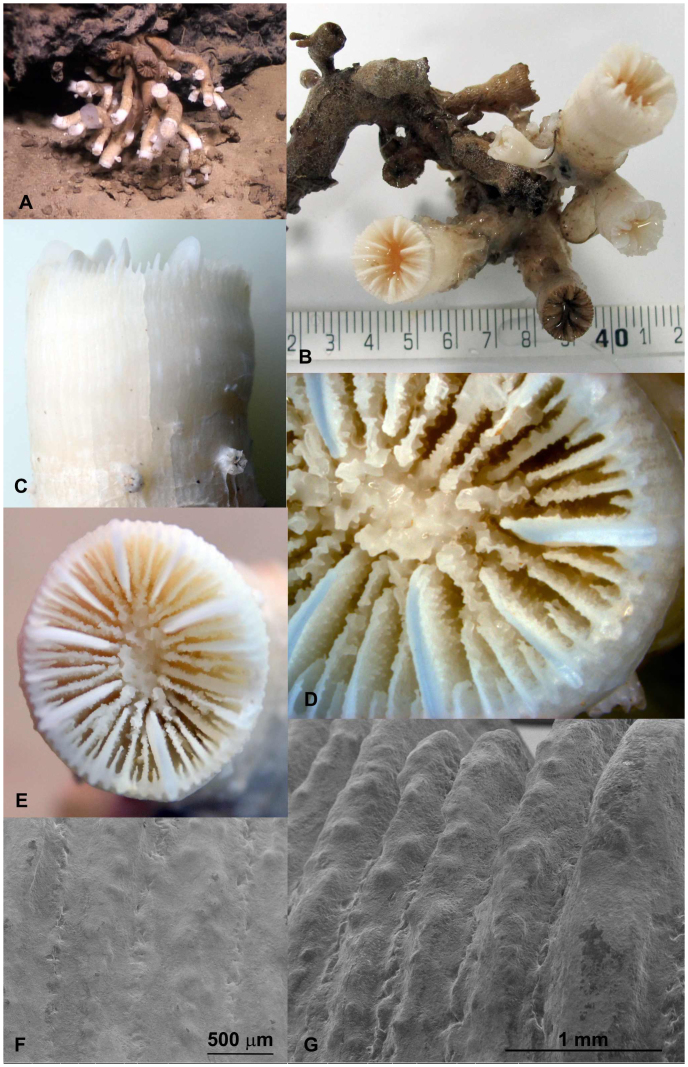
Species A (undetermined), Caryophylliidae. (a) Habitat view. (b) Colony view (scalebar in cm). (c) Lateral view of corallum. (d) and (e) Calicular view of corallum. (f) and (g) Close-up (SEM) of the theca half way up the corallum and at the top of the corallum respectively. Image a obtained with ROV camera. Image b by Roder/Voolstra. Images c, d, e, f, g by Bouwmeester.

Undetermined species of Caryophyllidae (hereafter “species A”) (Fig. 4): Corallum colonial, branching extra-tentacular, septal symmetry hexameral, septa arranged in 5 cycles, columella present, theca smooth with single row of bumps on ridge of costae, septa edges smooth, septa walls granular, pali present before septa of 3^rd^ cycle. Colonies were mostly observed on the bottom of rocky structures or ridges just above the sand, with a few colonies growing directly on the compacted sand.

The other three deep-sea coral species observed (Supplement Fig. 2) were not collected. One species, a single white solitary coral, observed within a field of dead skeletons of *Eguchipsammia fistula* at site 2 (Supplement Fig. 2a) could be determined as *Rhizotrochus typus* due to its funnel-shaped corallite, approximately 1 cm thick at the base and 5 cm wide at the top. On ridges at site 1, more solitary polyps were observed, attached to the hard substrate. An orange taxon was found (Supplement Fig. 2b–c) growing on the wall of the ridge and a white taxon was observed (Supplement Fig. 2d) hanging from the top of the ridge. Although HD video images were available, their resolution was still too low to identify either to family level or further.

**Figure 5:**
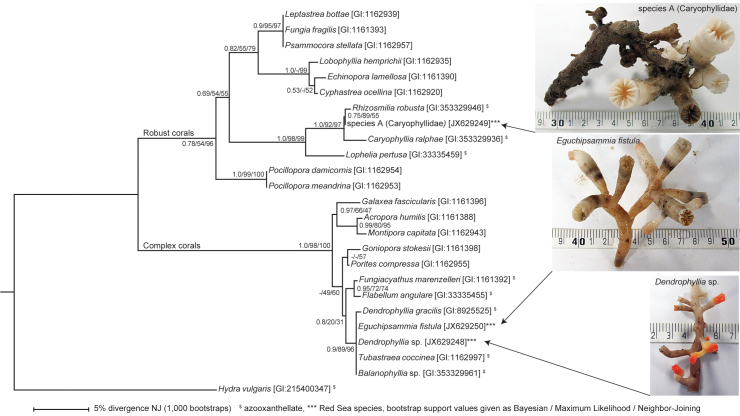
Molecular phylogram of relations among 24 species of corals and *Hydra vulgaris* (outgroup) as determined by Bayesian, Maximum Likelihood, and Neighbor-Joining analyses. This phylogram was generated on the basis of sequences from the mitochondrial 16S ribosomal gene region^26^,^27^,^28^. The numbers on the branches represent values from 1,000 bootstrap replicates presented in the order of Bayesian/Maximum Likelihood/Neighbor-Joining. Azooxanthellate species are denoted by ^$^, Red Sea species by ***. NJ: Neighbor-Joining. Images by Roder/Voolstra.

Phylogenetic analyses (Fig. 5) of partial sequences of the mitochondrial 16S ribosomal RNA encoding gene were conducted to confirm the taxonomic classification of our deep-sea coral specimens^22^,^25^. Morphological classifications coincided with molecular phylogenetic comparisons with published scleractinian coral sequences^26^,^27^,^28^. In the phylogenetic tree, all three species clustered with known cold-water and/or azooxanthellate corals. Furthermore, *E. fistula* and *Dendrophyllia* sp. could not be individually resolved but clustered with high bootstrap support with other members of the family Dendrophylliidae within the complex corals. Species A grouped with high bootstrap support with other members of the family Caryophyllidae within the robust corals.

### Metabolism

**Figure 6:**
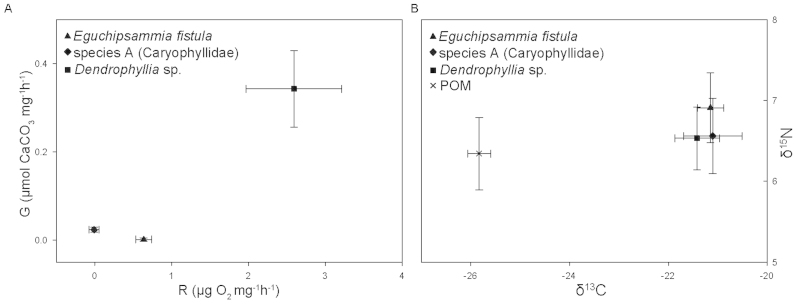
(A) Calcification (G) and respiration (R) rates (± SE) of three coral species found in the deep Red Sea (*Eguchipsammia fistula*, species A (Caryophyllidae), *Dendrophyllia* sp.). (B) Stable isotope measurements of δ^13^C and δ^15^N in the particulate organic matter (POM) fraction of the total suspended material in reef water from the Red Sea and of the tissue of the three coral species found in the Red Sea.

Shipboard-based incubations of the corals displayed an overall low metabolic activity (Fig. 6A). Respiration rates were not detectable for species A (Caryophyllidae) and lower for *E. fistula* than for *Dendrophyllia* sp. Similarly, calcification rates were also reduced in species A and *E. fistula*. Subsequent analyses of isotopic carbon and nitrogen ratios in the coral tissues (Fig. 6B) revealed similar ratios for all three species and both elements. While nitrogen values of particulate organic matter (POM > 0.7 μm) collected from 1 L of water in reef vicinity were similar to those of corals, carbon ratios were much lower in POM compared to corals (nearly 5‰).

## Discussion

In accordance with what is known from the presence of deep-sea scleractinian corals elsewhere^2^,^8^,^29^, deep-sea corals in the Red Sea were exclusively encountered in areas displaying strong topography such as steep walls or seamounts and attached to or associated with hard substrate, i.e. overhangs, rocks, edges, or faults. However, there are also striking differences between described habitats of deep-sea corals elsewhere and those where deep-sea corals occur in the Red Sea. The physical and biogeochemical properties of Red Sea habitats fall well outside established ecosystem boundaries for deep-sea corals in regard to temperature, oxygen availability, and salinity (Fig. 1, Table 1). Temperatures in the deep Red Sea are as high as in some tropical shallow reef communities and nearly 10°C higher than the next-warmest known habitat of deep-sea corals^2^. Additionally, salinity levels in deep-sea reefs of the Red Sea are higher than the highest previously recorded salinity for deep-sea coral communities (38.8 PSU)^6^. Studies of reefs built by *Lophelia pertusa*, perhaps the most well-studied deep-sea coral, have documented oxygen concentrations 3 to 5 times higher than what we recorded (ranging between 5.7 – 10.3 mg l^−1^)^29^. Most importantly, oxygen concentrations below 4 mg l^−1^ (3 ml l^−1^) have been shown to inhibit aerobic metabolism in deep-sea corals, and accordingly deep-sea corals in the Red Sea must have evolved adaptations to cope with these low levels, e.g. a highly reduced gross metabolic rate^18^. As atmosphere-segregated deep-sea water is rich in dissolved inorganic carbon compounds (resulting from remineralization processes^13^) cold-water corals typically exist in locations that are near the lower limits of aragonite saturation tolerance for calcifying organisms (undersaturation is defined as Ω = 1.0)^14^. In contrast, alkalinity and aragonite saturation levels in the Red Sea are high, even at greater depths and potentially support coral calcification. Changes in aragonite saturation can have profound impacts on marine calcifiers (including corals)^14^ and might be as important as temperature, especially in regard to global climate change. Factors influencing carbonate chemistry in the Red Sea might be the high salinity^30^, the warm temperature^13^, or a combination of the two and possibly other parameters. Because mechanisms driving aragonite saturation are complex especially in continental shelf or marginal seas^31^, it remains difficult to speculate whether unexplored habitats with similar conditions exist outside the Red Sea that might harbor as-yet undetected deep-sea corals.

Supporting the hypothesis that food availability is low, we observed very low abundances of other deep-water filter feeders common in temperate waters: Only few specimens of anemones and sponges (in *E. fistula* coral gardens), or sponges and octocorals (attached to rocks in the habitats of Species A and *Dendrophyllia* sp.) could be observed at our study sites. Indeed, concentrations of inorganic nutrients and suspended matter in subthermocline waters of the Red Sea are lower than in temperate oceans, the latter with decreasing quality over depth (i.e., nitrogen becomes quickly depleted compared to organic carbon) as would be expected for decaying organic material^32^. Moreover, the relatively low primary production in Red Sea waters^19^ seems at odds with the assumption that deep-sea coral nutrition is highly dependent on food transport from productive surface waters to less productive deep waters^4^.

Of the three collected coral species only one (*Eguchipsammia fistula*) could be identified to the species level. For the two other species (*Dendrophyllia* sp. and species A [Caryophyllidae]) we were not able to determine the specimens to the species level. We consistently had exclusive encounters of all species in a distinct microhabitat indicative of niche partitioning. *E. fistula* forms monospecific coral gardens on seamount tops. Species A is found on hard or sandy substrates close to rocky structures. We cannot exclude that corals were originally attached to nearby rocks and continued to grow on the soft bottom after breaking off, or that the hard substrate originally used for settling became gradually covered with a fine layer of sediment and therefore was not visible. *Dendrophyllia* sp. was found mainly attached upside down from hanging walls. It remains to be determined whether or how these distinct niches relate to adaptations for living in the challenging conditions in the deep Red Sea.

*E. fistula*, among the first deep-sea corals found in the Red Sea^22^, is also known to occur in the Indo-Pacific, Australia, and New Zealand^33^. This indicates that larval connectivity between the Red Sea and the Indian Ocean exists (or existed), and that *E. fistula* is or was connected via the Bab-el-Mandeb^34^. Genetic connectivity studies between specimens from the Indo-Pacific and Red Sea should help to verify this observation.

Deep-sea corals in the Red Sea show specific adaptations with very low respiration and calcification rates in comparison to, e.g., *L. pertusa*, which has a respiration rate of about 0.5 μmol g^−1^ h^−1^ 18 and a calcification rate of up to 1% of its entire weight per day^35^. Interestingly, in all species analyzed, only polyps were alive (i.e., the distal end of the corals), whereas the rest of the skeleton was not covered with tissue. This might be one of the adaptations to living in warm and deep waters by minimizing metabolic needs. Higher respiration rates of *Dendrophyllia* sp. compared to the two other species might be explained with regard to their habitat. It was mainly encountered hanging from rocks into the open water column, whereas species A and *E. fistula* were found to be bottom dwellers with likely less exposure to resources, and hence with greater food and oxygen depletion. Similarly, calcification rates were low in all species despite the high aragonite saturation levels, but even lower in species A and *E. fistula* in comparison to *Dendrophyllia* sp. This indicates that other factors, such as oxygen supply, might limit the growth of deep-sea corals in the Red Sea. It will be interesting for future studies to determine age and growth rate of deep-sea corals of the Red Sea. Our initial results suggest that deep-sea corals in the Red Sea seem to compensate for the lack of nutrients and oxygen through greatly reduced calcification and respiration rates.

Interestingly, isotopic signatures of coral tissue and particulate organic matter (POM) indicate that deep-sea corals in the Red Sea do not exclusively feed on POM present in the vicinity of the reef. Nitrogen signatures have been shown to be enriched by 2.5–3.5‰ in *L. pertusa* and *Madrepora oculata* compared to local particulate organic matter (POM)^36^, whereas nitrogen signatures in our coral specimens were only slightly enriched (by about 0.5‰). It might be possible that deep-sea corals in the Red Sea utilize plankton not captured in the POM fraction as a food source (Red Sea euphotic zone plankton have a nitrogen signature of about 4.5‰^37^) as the Red Sea is known to have extraordinary zooplankton migrations with a subsurface zooplankton maximum between 100–750 m^38^. However, carbon signatures recorded for the same Red Sea plankton ranged from −17 to −20‰^37^ and as carbon levels are known to increase by about 0.5–1.0‰ per trophic level^39^, the shift in our carbon data suggests that yet other food sources (or processes, e.g. microbial) might contribute to the corals' diet. Moreover, a study on trophic food webs in the deep sea found intricate and distinct trophic pathways, i.e. observed large overlaps in isotopic signatures between the different food web members were discussed as a result of competition for and adaptation to limited food availability^40^. Considering the potential complexity of such deep-sea food webs, further work in the Red Sea system will be needed to unravel the nature of the trophic pathways these corals utilize.

In summary, the existence of deep-sea coral communities in the warm, saline, oxygen- and food-deprived environment of the Red Sea extends ecosystem-boundaries defined by current knowledge. We propose that the corals' survival is a combined result of maximizing available resources while minimizing metabolic demands. They appear to maintain minimal amounts of tissue to survive and have strongly reduced respiration rates, which may be some of the key adaptations to meet the metabolic challenges of low oxygen and high temperature. Calcification is facilitated under conditions of high temperature and aragonite saturation, but it may be reduced in deep-sea corals in the Red Sea as a consequence of low oxygen levels and scarce food availability.

Our findings suggest that existing theories on deep-sea coral ecology need to be re-examined. Clearly, not all species are dependent on cold-water biotopes. Rather, the deep-sea corals of the Red Sea expand the known physical and chemical boundaries of deep-sea corals and provide new insights into understanding environmental constraints and controls in these ecosystems. The Red Sea constitutes a marginal habitat that allows for the study of adaptation of corals that thrive in cold, deep waters (e.g., the Indo-Pacific) as well as the warm and deep waters of the Red Sea. Understanding the ability of these ecosystems to adapt and function is paramount given that global climate change is significantly altering natural environments at an unprecedented rate.

## Methods

### Study sites

During an expedition (December 1 - 13, 2011) on the R/V Aegaeo (operated by the Hellenic Center for Marine Research, Greece) in the central Red Sea, we explored four sites (Table 1, Figure 1). The sites were characterized using various combinations of approaches (detailed in Table 1): bathymetric seafloor mapping (Seabeam 2100), conductivity-temperature-depth (CTD) profiling, water sampling, a remotely operated vehicle (ROV) (Max Rover), and a manned submersible (THETIS, Comex S.A.)

Multibeam sonar was used to identify specific sites with suitable topographic relief (i.e., seamount-like or steep-walled habitats) as candidates for further investigation using the ROV and submersible.

ROV video transects were recorded using a SEA MAX universal underwater video camera with pan and tilt and three modules (two wide angle and one zoom color video features). This camera was deployed to record continuously. We also used two High-Definition Ocean ProHD Undersea cameras with pan and tilt (with wide angle and zoom features), which were utilized only at sites of interest. The manned submersible was similarly equipped with a high-resolution video camera (XC 999 P). Subsequent video analyses identified characteristics of deep-sea coral habitats. A total of 33 hours of video footage was analyzed.

### Environment of the deep Red Sea

A CTD (SBE 37-SMP) attached to the ROV monitored temperature in the sampling areas at all times, while a Niskin was entrained three times at each site to the ROV to sample water directly from the benthic boundary layer for measurements of total alkalinity (TA) and aragonite saturation (Ω_arag_), inorganic nutrient (nitrate + nitrite), and total suspended matter (TSM) contents, as well as stable isotope ratios (δ^13^C and δ^15^N) of the organic fraction of the suspended material (details on methodology below).

For each Niskin, 50 ml of water were carefully drained into sampling containers and immediate analyzed. Ω_arag_ was calculated from TA, pH, temperature, and salinity using the CO2SYS Excel program^41^. Another 1 l of water from the Niskin was filtered (200 mm Hg) on pre-combusted and pre-weighed filters (Whatman GF/F), which were stored at −20°C until being lyophilized. TSM was determined (to 0.01 mg) on a microbalance (Mettler Toledo). The filters were cut in half; while one half was acidified (0.1 N HCl) to remove inorganic carbon for the determination of the stable isotope ratio of organic carbon (δ^13^C), the other half was used for determination of the nitrogen isotope ratio (δ^15^N).

Isotope ratios were quantified with an isotope ratio mass spectrometer (Delta plus XP, Thermo Finnigan) relative to Pee Dee Belemnite (PDB) standard and atmospheric nitrogen for carbon and nitrogen, respectively. As the exact weight of the sample was determined prior to analysis, the ratio of organic carbon and nitrogen (CN) could be determined via the coupled elemental analysis (yielding the weight percent of carbon and nitrogen) during the isotope measurements. Inorganic nutrient concentrations of the filtrate (frozen at −20°C until analysis) were determined using standard colorimetric tests in an autoanalyzer (QuickChem 8000, Zellweger Analytics Inc.).

In March 2012, we sampled the water column between sites 1 and 2 for physical and biogeochemical analyses. We repeatedly lowered a CTD (Ocean Seven 320 Plus, Idronaut) including probes for pH and oxygen and recorded measures of depth, temperature, salinity, oxygen, and pH between 0 and 800 m in one-second intervals. Additionally, water samples were collected over the same depth range via Niskin deployment (3 times) for subsequent analyses (all methods as above). TSM and corresponding CN ratios were measured for each two replicates at 1, 5, 20, 50 (only one replicate), 100, 200 (only one replicate), 300, 500, 600, 700, 800 m. Inorganic nutrients (nitrate + nitrite) were measured in triplicates at 5, 20, 250, 500 (only two replicates), 600 (only two replicates) m. TA and Ω_arag_ were measured in triplicates at 5, 50, 100, 200, 300, 400, 500, 600 (only 2 replicates), 700, 800 m.

In May 2012, light penetration of the upper 300 m of the water column was measured at the same locations using optical sensors (RAMSES ACC-VIS, TriOS) to determine the maximum boundaries for light-dependent communities. In total, three casts were conducted and light spectra between 270 and 1000 nm wavelengths were measured at every 5 m. The boundary of the photic zone was determined as the depth beyond which the respective wavelength was less than 1% of the sunlight measured at the surface.

### Species observed

Deep-sea corals that were observed were classified to the highest taxonomic resolution possible. Some samples were collected using the manipulator arm of the ROV and a sampling basket (Table 1) and were subsequently morphologically examined. One specimen from each collected species was bleached in sodium hypochlorite for 12 hours to remove organic matter. Samples were further assessed using traditional microscopy and scanning electron microscopy (SEM) and identified to the closest taxon. The skeleton samples were left uncoated for analysis by SEM on a Quanta 200 FEG SEM at low vacuum. *Eguchipsammia fistula* was identified according to descriptions from Alcock^42^ and Marenzeller^22^, whose taxonomy was later updated by Cairns^43^. *Dendrophyllia* sp. was identified with the help of Cairns^25^ and species A (Caryophylliidae) was run through an identification key to the genera of azooxanthellate scleractinia^44^, but could not be identified further than the family level as morphological characters did not further match any of those of the key, and no species or genera descriptions from various earlier expeditions matched our samples. *Rhizotrochus typus* was identified according to Scheer and Pillai^45^. For other non-collected corals we can only report limited taxonomic resolution based on video imagery.

We also utilized molecular approaches to determine taxonomic relationships where possible (samples evaluated after incubations, details of sample handling in next paragraph). From the three species we collected, DNA was extracted using the Qiagen DNeasy Plant Mini Kit following instructions from the manufacturer. 10 – 30 ng of DNA was used in a 30 μl PCR reaction using the Qiagen Multiplex PCR kit and primers LP16SF/LP16SR^27^. The temperature cycling for amplification were 1 cycle at 95°C for 15 min; 30 cycles at 95°C for 30 sec, 55°C for 90 sec, and 72°C for 90 sec; and one final extension at 72°C for 30 min. PCR products were subsequently purified using the Qiagen MinElute PCR purification kit according to manufacturer's instructions, cloned into Qiagen pDrive, and sequenced on an ABI 3730 XL. Sequences were clipped, trimmed, and assembled with CodonCode Aligner. All assemblies yielded single contigs that are accessible in GenBank: *Eguchipsammia fistula* [JX629250], *Dendrophyllia* sp. [JX629248], species A (Caryophylliidae) [JX629249]. Alignment of partial mitochondrial 16S ribosomal RNA genes was generated with MUSCLE as implemented in MEGA5^46^. All sites with missing data were deleted, which yielded 247 characters. Neighbor-Joining and Maximum Likelihood trees were generated with MEGA using the T92 substitution model and a discrete gamma distribution with 5 categories as determined from the model test function implemented in MEGA. Bayesian analysis was performed with MrBayes^47^ and the nst = 1 substitution model using a discrete gamma distribution and 5 categories. MrBayes was set to run until convergence using the stop rule and a value of < 0.01 for the standard deviation of split frequencies. 1,000 bootstrap replicates were conducted for all analyses.

### Metabolic measurements

We collected specimens of three species (Table 1). 5 samples per species were assessed in aquaria onboard the ship after collection. After acclimatization of a minimum 4 hours in opaque tanks filled with freshly collected seawater, we incubated each specimen in 1 l beakers for 60 – 80 minutes. The running of the vessel engine ensured a constant moderate stirring of the water in the tanks and beakers. The temperature in the tanks was monitored and remained constant at around 21 ± 0.5°C. Control incubations were run with beakers containing only water. Before and after incubation, the oxygen content of the beakers was measured using an oxygen probe (Multi 3500i, WTW). Water from the beakers was also sampled for total alkalinity using the Gran approximation by determining the second endpoint^48^ via automatic potentiometric titration (using 0.01 N HCl) on a titration apparatus (Titrando 888, Metrohm). Calcification rates were derived via the alkalinity anomaly technique^49^ based on the 2:1 stoichiometric relationship between TA and CaCO_3_ using automatic potentiometric titration. Changes in the control incubations were less than 0.05% and therefore not included in subsequent calculations. Respiration and calcification rates were obtained by subtracting values obtained after incubation from values obtained before incubation.

After incubation, coral samples were flash-frozen in liquid nitrogen and the polyps were ground to a fine powder for tissue analyses. An aliquot (approximately 3 mg) was acidified with 0.1 N HCl to remove inorganic carbon for the determination of the stable isotope ratio of organic carbon (δ^13^C); another aliquot of the same amount was directly used for the measurement of organic nitrogen (δ^15^N).

## Author Contributions

M.L.B., C.R., C.R.V. conceived the research cruise. C.R. and C.R.V. designed and conducted the metabolic and isotopic measurements. C.R., C.R.V., J.B. generated data. C.R., C.R.V., J.B., M.L.B. wrote the article, analyzed and interpreted data. E.P. and A.A.S. provided materials, reagents, and logistical support. All authors critically read the article and approved the final version.

## Supplementary Material

Supplementary Information Supplement

## Figures and Tables

**Table 1:** Summary of sites, locations, species observed, environmental characteristics and other measurements at four study sites in the central Saudi Arabian Red Sea. Measurements are listed with (±SE). GenBank accession numbers are included with each species. TSM: Total Suspended Matter, OC: Organic Carbon. Note that at all sites bathymetric data, video recordings, and CTD data were collected

Site #	Geographic Location	Species	GenBank ID	Depth [m]	Habitat	# obeserved along ROV track	Temp (°C)	Oxygen (mg l^−1^)	Ω_arag_	TSM (mg l^−1^)	OC in TSM (wt.%)	Nitrite + Nitrate (μmol l^−1^)	sampled	metabolic measurements	water samples
1	22°46.025′N/38°03.564′E	Sp A (Caryophyllidae)	[JX629249]	600–700	on hard substrate, sometimes on sandy bottom and close to rocky structures	> 14	21.53 ± (0.0001)	1.18 (±0.002)	3.48 (±0.02)	1.47 (±0.08)	75.65 (±11.76)	16.32 (±0.13)	Y	Y	
		*Dendrophyllia* sp.	[JX629248]	570–640	associated with ridges, mainly hanging	8	21.53 (±0.00006)	1.54 (±0.00004)	3.44 (±0.03)	2.07 (±0.43)	70.04 (±7.38)	15.47 (±0.56)	Y	Y	Y
		Red polyp (solitary)		680–740		5							N	N	
		White polyp (solitary)		600–740		7							N	N	
2	22°14.862′N/38°53.598′E	Sp A (Caryophyllidae)		240–550		7							Y	Y	
		*Rhizotrochus typus*		280–290		1							N	N	Y
		*Eguchipsammia fistula*	[JX629250]	230–270	forming monospecific coral gardens on seamount tops	> 100	21.60 (±0.0006)	1.02 (±0.004)	3.61 (±0.06)	1.52 (±0.09)	75.16 (±13.16)	16.58 (±0.21)	Y	Y	
3	22°16.328′N/38°49.071′E	Sp A (Caryophyllidae)		560–590		1							N	N	N
4	22°17.835′N/38°53.815′E	*Eguchipsammia fistula*		320		> 50							Y	N	N
