# Supplementary material for: First biological measurements of deep-sea corals from the Red Sea
Source: Sci Rep. 2013 Oct 3;3:2802. doi: 10.1038/srep02802 (PMC3789407; doi:10.1038/srep02802)
Supplement: Supplementary Information — Supplement [file srep02802-s1.doc]

First biological measurements of deep-sea corals from the Red Sea

C. Roder^1*^, M. L. Berumen^1,2^, J. Bouwmeester^1^, E. Papathanassiou^3^, A. Al-Suwailem^4^, C. R. Voolstra^1*^

^1^Red Sea Research Center, King Abdullah University of Science and Technology, Thuwal, Saudi Arabia

^2^Biology Department, Woods Hole Oceanographic Institution, Woods Hole, USA

^3^Hellenic Centre for Marine Research, Anavissos, Greece

^4^Coastal and Marine Resources Core Lab, King Abdullah University of Science and Technology, Thuwal, Saudi Arabia

*Correspondence to: cornelia.roder@kaust.edu.sa, christian.voolstra@kaust.edu.sa.

Running title: Biology of Red Sea deep-sea corals

**Supplement Fig. 1:** Map of study area and specific study sites for deep-sea corals in the central Red Sea. Insets show multibeam bathymetry where further surveys were conducted by ROV and manned submersible. Depth (m) is indicated by the color gradient as shown in the key. The grey line indicates the route followed by a remotely operated vehicle (ROV) for detailed habitat surveys and sample collections.

The background map was created with ArcGIS 10.1 (Esri). Bathymetry maps were created with Fledermaus 7 Visualization and Analysis Software (Ageotec) and ArcGIS 10.1 (Esri). Editing was done using Adobe Illustrator CS3 13.0.0 and Adobe Photoshop CS3 10.0.


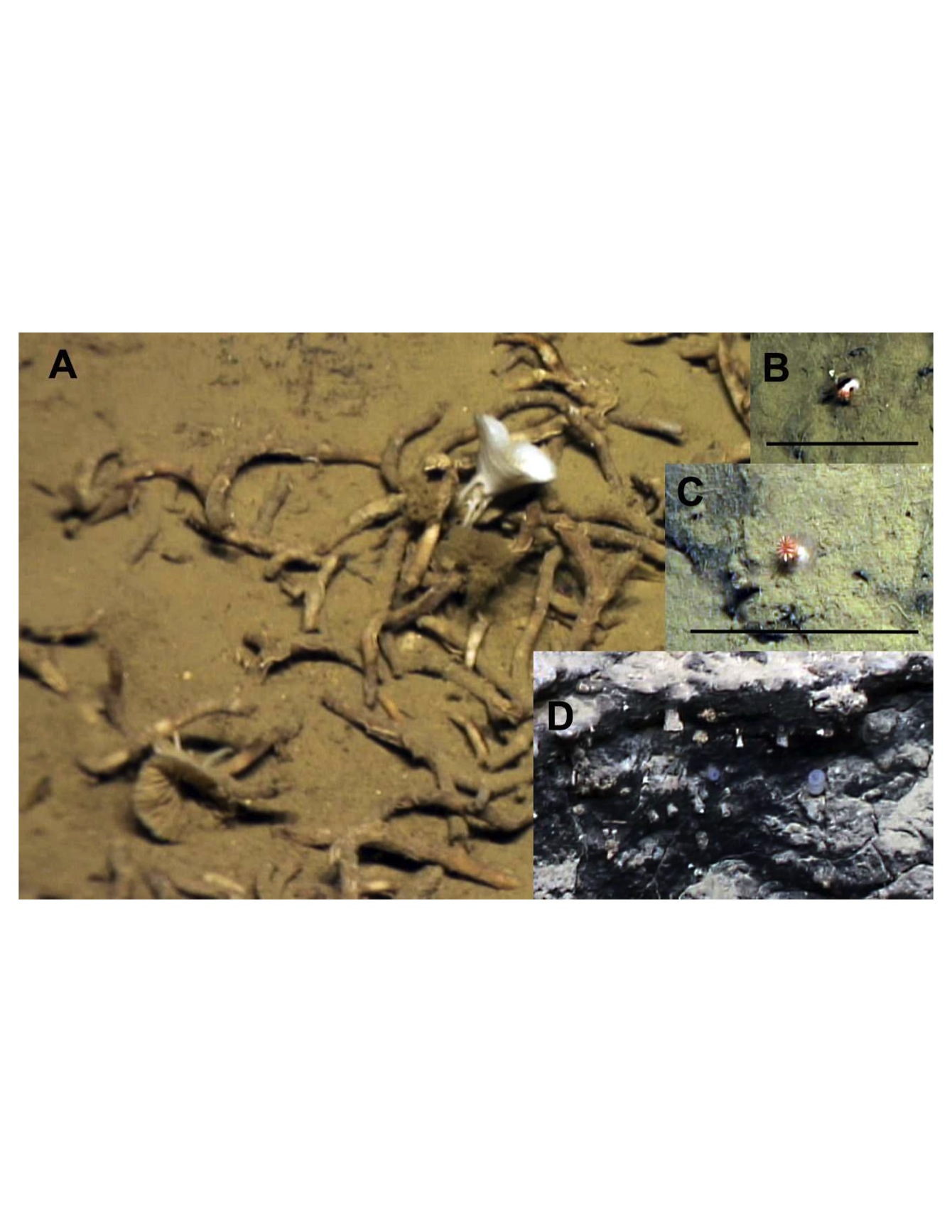
**Supplement Fig. 2:** Images (best resolution available) extracted from the ROV HD video recordings of other specimens observed. **a** *Rhizotrochus typus* in a field of dead skeletons of *Eguchipsammia fistula* (see Fig. 3), live specimen top-right and dead specimen bottom-left. **b** and **c** Orange solitary coral attached to a seamount wall. **d** White solitary corals hanging under a ridge.
